# Pharmacokinetic properties of a novel formulation of *S*-adenosyl-l-methionine phytate

**DOI:** 10.1007/s00726-021-03076-7

**Published:** 2021-09-18

**Authors:** Antonio Francioso, Sergio Fanelli, Maria d’Erme, Eugenio Lendaro, Niccolò Miraglia, Mario Fontana, Rosaria A. Cavallaro, Luciana Mosca

**Affiliations:** 1 Department of Biochemical Sciences A. Rossi Fanelli, Sapienza University, Rome, Italy; 2 Department of Medical‑Surgical Sciences and Biotechnologies, Sapienza University, Latina, Italy; 3 Clinical and Pre-clinical Development, Gnosis by Lesaffre, Milan, Italy; 4 Department of Surgery P. Valdoni, Sapienza University, Rome, Italy

**Keywords:** *S*-adenosyl-l-methionine, Pharmacokinetics, Rat, HPLC, Phytate, Tosylate

## Abstract

*S*-adenosyl-l-methionine (SAM), the main endogenous methyl donor, is the adenosyl derivative of the amino acid methionine, which displays many important roles in cellular metabolism. It is widely used as a food supplement and in some countries is also marketed as a drug. Its interesting nutraceutical and pharmacological properties prompted us to evaluate the pharmacokinetics of a new form of SAM, the phytate salt. The product was administered orally to rats and pharmacokinetic parameters were evaluated by comparing the results with that obtained by administering the SAM tosylated form (SAM PTS). It was found that phytate anion protects SAM from degradation, probably because of steric hindrance exerted by the counterion, and that the SAM phytate displayed significant better pharmacokinetic parameters compared to SAM PTS. These results open to the perspective of the use of new salts of SAM endowed with better pharmacokinetic properties.

## Introduction

**Fig. 1:**
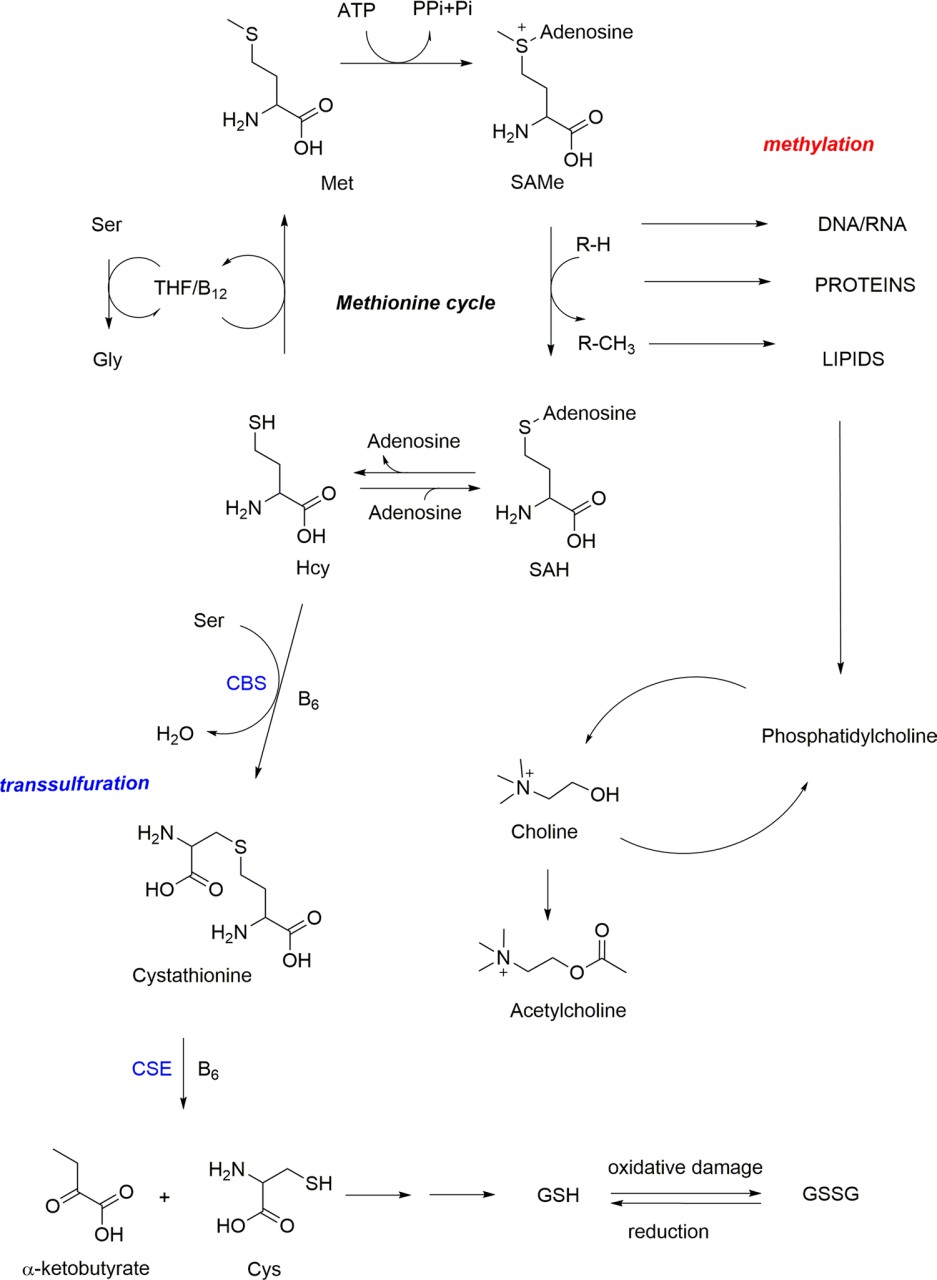
SAM methyl cycle and transmethylation/transsulfuration related pathway. Methionine is activated by ATP to produce SAM. The transfer of the methyl group from SAM by methyltransferase yields SAH, which is hydrolyzed to adenosine and Hcys. Hcys can be remethylated to regenerate Met by two Hcys methyltransferases, met synthase and betaine-Hcys methyltransferase (BHMT), or be used to synthesize cysteine by transsulfuration pathway reactions (CBS and CSE). Cys formed via transsulfuration pathways serves for biosynthesis of GSH. Phosphatidylcholine is synthesized through successive SAM-dependent methylations of phosphatidylethanolamine. Acetylcholine is synthesized by acetylCoA and choline formed by phosphatidylcholine hydrolysis. *Cys* cysteine, *Hcy* homocysteine, *SAH* *S*-adenosylhomocysteine, *SAM* *S*-adenosylmethionine, *Met* methionine, *Ser* serine, *Gly* glycine, *GSH* glutathione, *GSSG* oxidized glutathione, *CBS* cystathionine beta-synthase, *CSE* cystathionase

S-adenosyl-l-methionine (SAM) is an endogenous metabolite present in all living organisms and synthesized from methionine and adenosine triphosphate (ATP). It is the main biological methyl donor in transmethylation reactions of substrates like proteins, lipids, neurotransmitters and RNA and DNA and is involved in different biochemical pathways like methionine cycle and transsulfuration, which are correlated to the so called one-carbon metabolism (Fig. 1).

Transmethylation is the transfer of methyl groups from methyl donors carrying one-carbon units, like SAM, to an acceptor molecule. Transmethylation could be considered a step of the methionine cycle, given that by this reaction SAM is transformed into SAH, which will be hydrolyzed by SAH hydrolase to Hcy, which will in turn be remethylated to methionine or will enter in the transsulfuration pathway.

This complex biochemical pathway, regulated by the presence of folate, vitamin B12 and B6, plays a role in several physiological processes representing the link between methylation metabolism, epigenetics maintenance, and redox defense (Finkelstein 1998, 2007; Lu 2000; Anderson et al. 2012; Fleming et al. 2012; Ducker and Rabinowitz 2017; Mosca et al. 2019; Amenyah et al. 2020). SAM appears to be useful in the treatment of mood disorders since it is also involved in the biosynthetic pathway of acetylcholine, epinephrine, melatonin, norepinephrine, dopamine, serotonin (Mischoulon and Fava 2002), and in sulfur amino acid homeostasis (Lu and Mato 2012; Mato et al. 2013).

SAM level decreases with aging and SAM deficiency leads to alterations in one-carbon metabolism and transmethylation impairment that are associated with the onset of osteoarthritis, liver diseases, cardiovascular diseases, depression, and neurodegenerative diseases such as Alzheimer’s disease (Finkelstein 2003; Lu and Mato 2012; Glier et al. 2014; Jung 2015; Murray and Jadavji 2019). Several studies encourage the use of exogenous SAM supplementation in pharmacological doses to restore one-carbon metabolism, particularly in liver and neurodegenerative diseases. In fact, exogenous SAM appears to be generally well tolerated and able to cross the intestinal wall, increasing its plasma concentrations: moreover, a supplementation enhances its cerebrospinal fluid levels, demonstrating that SAM is able to cross the blood brain barrier (Bottiglieri et al. 1990, 1994; Lu and Mato 2012).

SAM is worldwide commercially available both as a nutraceutical supplement and as a pharmaceutical product, depending on the different dosage and on different local laws (Bottiglieri 2002). Despite its high solubility, SAM is characterized by a low cellular permeability and a high chemical instability. To counteract the poor systemic bioavailability after oral administration (0.5–1%) and its rapid metabolism, the use of exogenous high doses is necessary (Yang et al. 2009). In vitro cellular uptake studies on Caco-2 cell cultures demonstrate how the poor oral bioavailability shown by SAM can be correlated to absorption problems of the molecule, rather than to its rapid metabolism (McMillan et al. 2005). As a matter of fact, studies have been made to identify more stable SAM salts formulations containing large anions and in improving SAM absorption by the enteric system, even by in situ enzymatic synthesis (Morana et al. 2000; Thomsen et al. 2013).

**Fig. 2:**
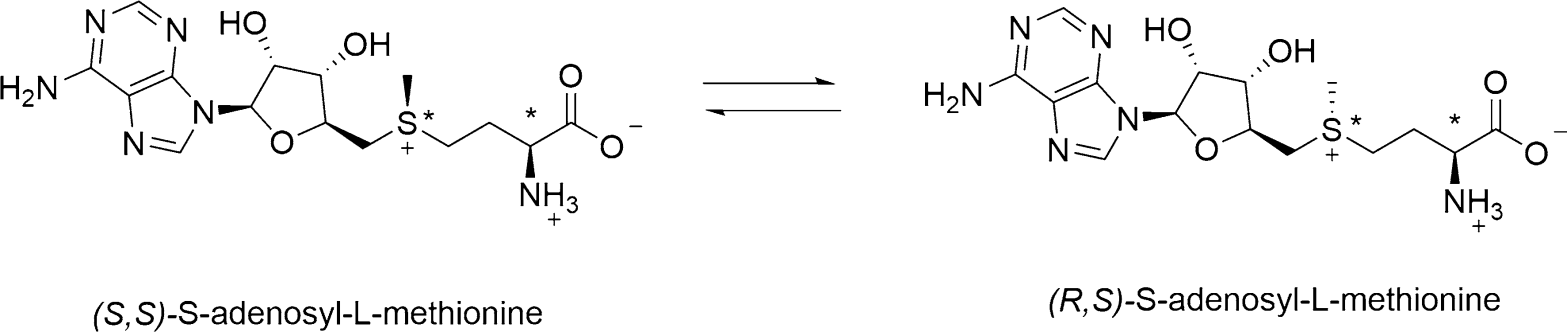
*S*-adenosyl-l-methionine diastereoisomeric equilibrium. Stereogenic centers are marked with an asterisk (*). *S*,*S* stereoisomer (left) is the biologically active form of SAM, which tends to isomerize leading to inversion of the configuration with equilibration

SAM exists in two diastereoisomeric forms: (*S*,*S*)-*S*-adenosyl-methionine and (*R*,*S*)-*S*-adenosyl-methionine, being only *S*,*S*-SAM the biologically active isomer (Fig. 2). SAM is produced by living organisms as a single diastereoisomer, *S*,*S*-SAM. In formulations, *S*,*S*-SAM tends to isomerize until the conditions of equilibrium with the two diastereoisomers in the 1:1 ratio are reached (Hoffman 1986; Desiderio et al. 2005). Moreover, one of the main concerns of SAM formulations is their strong chemical instability; SAM breaks down rapidly even at room temperature and specific pH values, giving rise to homoserine lactone, methylthioadenosine (MTA) and adenine and *S*-ribosyl methionine. SAM stability is improved when the molecule is presented as the salt of a strong acid. Several salts of SAM with strong organic or inorganic acids, including polyacids, are known, however, only sulfuric acid, 1,4-butanedisulfonic acid, and *p*-toluenesulfonic acid (tosylated) (PTS) salts are present on the market.

To find salts conferring a higher stability to SAM is still an issue. In this study a new SAM formulation consisting of a salt of SAM with phytic acid, a natural strong acid, has been employed. Phytic acid, also known as inositol hexaphosphate is present in plant seeds, where it functions as the main storage form of phosphorus. Stability data have shown that the salt of SAM with phytic acid is chemically stable. For this reason, this formulation is potentially able to retain a biological efficacy over a long time. In this study, a comparison of the bioavailability of this SAM phytate with the more commonly used SAM PTS salt has been investigated in rats.

## Materials and methods

### Chemicals

Gradient grade methanol, acetonitrile and ultrapure water for UPLC, were purchased from Carlo Erba (Milan, Italy). All other chemicals and solvents were of analytical grade and were from Sigma–Aldrich (Milan, Italy).

### Test products

*S*-adenosyl-l-methionine Tosylate disulfate (SAM PTS) and *S*-adenosyl-l-methionine phytate (SAM phytate) were provided by Gnosis by Lesaffre (Desio, MB, Italy), as two different formulations. The two salts were provided as dried powders. Solutions were prepared just before administration to rats dissolving the powders in water softened by reverse osmosis.

### SAM stability tests

The stability of SAM phytate was studied following the protocol for Long Term Stability described in the ICH guidelines for pharmaceuticals products. SAM phytate and SAM PTS samples were kept in climatic chambers at 25 °C with 60% Relative Humidity (RH). Two SAM phytate and three SAM PTS preparations were incubated for stability. SAM was determined by HPLC at *t*0 and after 1, 3 and 6 months. The stability of the molecule is expressed as the percentage residual assay (*t*/*t*0%).

Stability tests on SAM were performed by HPLC using a Beckman apparatus consisting of a Beckman 116 pump and equipped with an automatic sampler Mod. 507 with 100 µl loop. The column was a Phenomenex Luna SCX 100 A, 5 µm (250 × 4.6 mm) and a spectrophotometric photodiode array detector mod 168 set to a wavelength of 260 nm was employed. The analysis was performed in isocratic conditions with a mobile phase consisting of 0.5 M ammonium formate, pH was adjusted to 4.0 with formic acid. The chromatographic run was performed at 20 °C, at 1.2 mL/min. In these conditions the retention time for SAM was 11.0 min. Total run time was 45 min. An eight-point calibration curve in duplicate was obtained either using SAM PTS or phytate standard and used for SAM quantitation.

### Pharmacokinetic study protocol

The aspects of the protocol concerning animal welfare were approved by RTC animal-welfare body. The animal treatment phase for this study was performed at the European Research Biology Center S.R.L (ERBC) facilities, former Research Toxicology Center (RTC), Pomezia, Rome, Italy. Procedures and facilities were compliant with the requirements of the Directive 2010/63/EU on the protection of animals used for scientific purposes.

Sprague Dawley rats, 7–9 weeks old and with body weight of approximately 170–200 g, were ordered from Envigo RMS srl, San Pietro al Natisone, Udine, Italy. The animals were housed in a limited access rodent facility for an acclimatisation period of approximately 4 weeks before the start of treatment. Animal room controls were set to maintain temperature and relative humidity at 22 ± 2 °C and 55% ± 15%, respectively. Drinking water was supplied ad libitum to each cage via water bottles. Commercially available laboratory rodent diet (4 RF 21, Mucedola S.r.l., Milan, Italy) was offered ad libitum throughout the study, except for an overnight fast prior to dosing and approximately 2 h after dosing. SAM was administered in fasting conditions to avoid possible interference of food consumption on SAM intestinal absorption, SAM absorption is higher in fasting conditions (Cameron et al. 2020). For this reason, rats were deprived of food the night before supplement administration, fasting was extended to the two hours following administration, when most of the absorption occurs.

The pharmacokinetic profile of SAM phytate, compared to SAM PTS, was investigated in rats after single oral administration, according to the following experimental design:

- Group 1 comprised 16 male and 16 female rats that were given 133.7 mg/kg of SAM phytate (test substance);
- Group 2 comprised 16 male and 16 female rats that were given 95.4 mg/kg of SAM PTS (reference substance).

Test and reference substances were dissolved in water and administered by gavage to each animal at a dose volume of 10 mL/kg. Since the substances were dissolved in water, no control group was deemed necessary. Rats were administered with equimolar solutions of SAM PTS and SAM phytate (125 μmol/kg of body weight). The following investigations were performed: clinical signs, body weight and blood sampling for pharmacokinetic profile, which was carried out as follows: pre-dose and 0.5, 1, 1.5, 2, 4, 8, and 24 h after dosing. At each sampling time, at least 0.8 mL of blood samples were collected from the tail vein of 4 males and 4 females per each sampling time. Each animal was sampled at a maximum of two alternating time points. Maximal care was taken to avoid haemolysis of blood samples. Samples were transferred into tubes containing EDTA anticoagulant and centrifuged at room temperature. The plasma was divided into two aliquots of 200 µL each. Samples were stored at − 80 °C until analysis. All samples were analyzed within 2 months.

Animals that had completed the scheduled test period were killed with carbon dioxide at the end of the last blood sampling procedure. No necropsy was performed and no organs were retained.

### Chromatographic determination of plasma SAM

The chromatographic method routinely used for the stability tests was not suitable to be used for plasma analyses, as plasma is a complex biological matrix. Hence to analyze SAM level in plasma we set up a specific chromatographic procedure. An aliquot of plasma (200 μL) was treated with 20 μL of 3,5 M perchloric acid. After 30 min incubation on ice, the samples were centrifuged at 15000*g* at 4 °C for 30 min. 160 μL of supernatant were analyzed by HPLC/DAD on a Waters apparatus consisting of Waters 60F pumps and 600 pumps control unit system equipped with a Symmetry C18, 5 μm, 4.6 × 250 mm column associated to a guard column of the same material (Waters Corporation, Milford, Massachusetts, USA), an inline degasser, a Waters 717 auto-sampler and a spectrophotometric photodiode array detector mod 2996. The mobile phase A consisted of NaH_2_PO_4_ 50 mM, pH 4.4, containing 10 mM heptansulfonic acid, while mobile phase B was methanol. Isocratic elution was performed with 85% A and 15% B at a flow rate of 1 mL/min at 25 °C with the spectrophotometric detector set at 259 nm. In these chromatographic conditions SAM showed a retention time of 8.7 min. SAM was identified in the biological specimens by its retention time, UV–Vis spectrum and by spiking with authentic standard. An eight-point calibration curve in duplicate was obtained either using SAM PTS or phytate standard. No differences were observed in the quantitation of the analyte between the two forms. The method showed a detection limit of 0.15 µM and a linearity in the range 0.15–30 µM. The instrument control and data acquisition were carried out using the Waters Millennium^32^ software.

### Pharmacokinetic and statistical analysis

The statistical analyses were basically descriptive. The standard methods used for the pharmacokinetic analyses were carried out. Data are presented as mean, standard deviation (SD) or standard error of the mean (SEM), median and interquartile range.

To evaluate the pharmacokinetic parameters, plasma concentrations of SAM were calculated as micromolar units (µM), by subtracting the basal level of SAM concentration in plasma ([SAM] in plasma at pre-dose time point). The dataset prepared with the original data were corrected as follows:

$${\text{Data baseline corrected}}\, = \,{\text{original data}}{-}{\text{baseline SAM value}}.$$

In case of negative values, as result of the above correction, the data were considered as zero.

The pharmacokinetic parameters considered for the analysis were the area under the concentration–time curve 0 to 8 h (AUC_0–8_), the area under the concentration–time curve 0 to 24 h (AUC_0–24_), the maximum of concentration (*C*_max_) and the time to maximum concentration (*T*_max_).

The *C*_max_, defined as maximum plasma concentration in the time point distribution, was calculated for each group of animals (4 males and 4 females per each sampling time) at each sampling time. For each group, the *C*_max_ was the Maximum baseline-corrected value found in each distribution. The *T*_max_ was the corresponding time point.

The total *C*_max_ was defined as the mean of the single *C*_max_ by group of animals. The total *C*_max_ is presented along with its standard deviation, median, and interquartile range (IQ—25th and 75th percentile). As for the *T*_max_, it was also calculated by subject and presented as mean (SD) and median [IQ range].

Starting from the analysis dataset, the trapezoidal method was adopted for the AUC (at time points 8 h and 24 h) calculation according to the following formula:

$$\left( {C_{n - 1} \, + \,C_{n} } \right) \, \times \, \left( {T_{n} - T_{n - 1} } \right) \, /2,$$

where *C* is the plasma concentration and *T* the time point.

In addition to the descriptive analyses proposed in the study protocol, to compare the results of the two groups at the *C*_max_ and the all the time points after, an inferential analysis was proposed. The comparisons were performed through the Wilcoxon test.

## Results

### SAM phytate stability

**Fig. 3:**
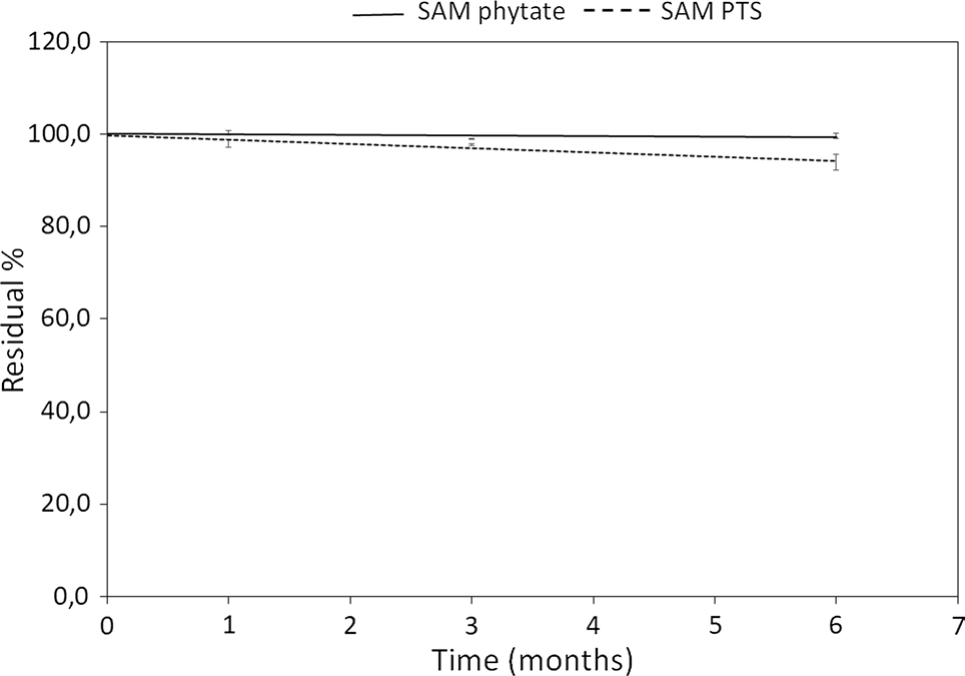
Residual assay (*t*/*t*0%) for SAM phytate (black line) and SAM PTS (dotted line). Values are expressed as mean values for each timepoint ± SD. Two SAM phytate preparations and three SAM PTS preparations were incubated in a climatic chamber set at 25 °C with relative humidity of 60% (according to ICH guidelines) up to 6 months. SAM was determined by HPLC before incubation (*t*0) and after 1, 3 and 6 months. The relative assay (*t*/*t*0%) was calculated

To assess the stability of SAM phytate in comparison with the more common SAM PTS, the only two available batches of the SAM phytate formulation and three SAM PTS preparations were incubated in a climatic chamber set at 25 °C with relative humidity of 60% (according to ICH guidelines) up to 6 months. The SAM was determined by HPLC before incubation (*t*0) and after 1, 3, and 6 months. The relative assay (*t*/*t*0%) was calculated and the resulting graphs of the mean values for both salts are reported in Fig. 3. SAM phytate showed to retain up to 99.6% of the initial SAM assay after 6 months, even better than PTS salt which showed a residual assay of 93.9%.

### Pharmacokinetics

#### Clinical signs

Throughout the study, all animals were checked early in the morning each working day and again in the afternoon. All clinical signs were recorded for individual animals. No unscheduled death occurred during the study. No clinical signs were recorded during the dosing and bleeding procedures in all animals. The animals showed a body weight in agreement with the age and sex for this species and strain.

### Pharmacokinetic analyses

**Fig. 4:**
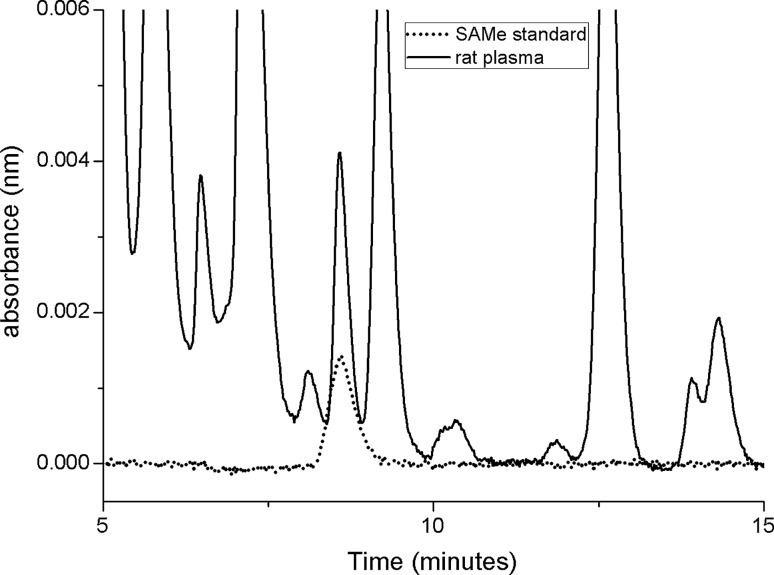
Chromatographic profiles of SAM PTS analytical standard (dotted line) or rat plasma (black line) after single oral administration. The chromatographic profile of the analytical standard was compared with that of plasma. SAM shows a retention time of 8.7 min, and was identified in the biological specimen by its retention time, UV–Vis spectrum and by spiking plasma with authentic standard

A representative chromatographic analysis on rat plasma samples is reported in Fig. 4, compared with a SAM standard sample.

The concentration of SAM was determined in plasma at the following times: 0 h (pre-dose Day 1), 0.5 h, 1 h, 1.5 h, 2 h, 4 h, 8 h, and 24 h (Day 2) after dosing. SAM basal level was found to be 0.34 µM. Pharmacokinetic variables examined, i.e., *C*_max_, *T*_max_, and AUC_0-*t*_ of SAM in plasma after single dose administration, were baseline-corrected.

**Fig. 5:**
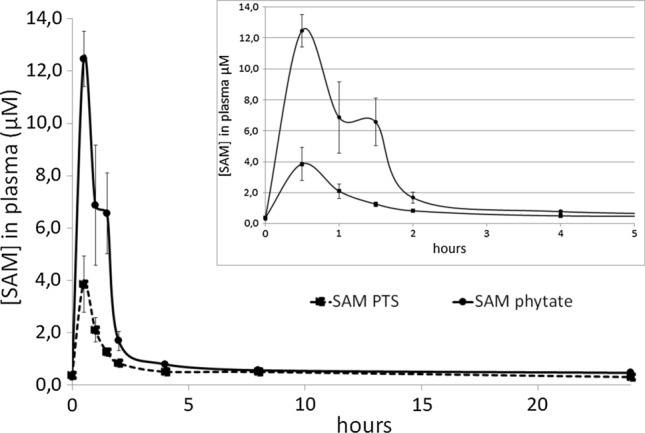
Mean plasma concentration–time profiles of SAM PTS (dotted line) or SAM phytate (black line) after single oral administration. Rats were administered with equimolar amounts of SAM phytate or SAM PTS via gavage. Plasma was sampled at 0.5, 1, 1.5, 2, 4, 8, and 24 h after dosing and SAM concentration in plasma SAM was determined by HPLC analysis. Data are mean ± SEM of 8 independent measures for each timepoint

The time course of mean ± SD plasma SAM concentrations after a single oral dose of SAM phytate and SAM PTS are shown in Fig. 5.

### *C*_*max*_* and T*_*max*_

The *C*_max_, defined as maximum plasma concentration in the time point distribution, was calculated for each animal and was the maximum baseline-corrected value found in each distribution. The *T*_max_ was the corresponding time point. The total *C*_max_ was defined as the mean of the single *C*_max_ by animal, which is different from the *C*_max_ shown in the curves of Fig. 4, i.e., the *C*_max_ of the mean values by time point. The total *C*_max_ is presented in Table 1.

**Table 1:** Pharmacokinetic parameters for the test and reference group

	Statistics	SAM phytate *N* = 8	SAM PTS *N* = 8
*C*_max_ (µM)	Mean ± SD Median (Q1–Q3)	13.37 ± 2.96 11.77 (11.41–15.5)	4.04 ± 3.00 2.76 (1.68–6.75)
*T*_max_ (hours)	Mean ± SD Median (Q1–Q3)	0.81 ± 0.37 0.75 (0.50–1.00)	0.75 ± 0.27 0.75 (0.50–1.00)

The mean (± SD) *C*_max_, calculated on the means of the single subject distributions, was 13.37 µM (± 2.96) in the SAM phytate group vs. 4.04 µM (± 3.00) in SAM PTS group.

As the *T*_max_ is expressed as time, the median (and IQ range) is the statistic to be considered. The median [IQR] *T*_max_ calculated on the single distributions was identical in both groups, i.e., 0.75 [0.50–1.00].

### Area under the curve (AUC)

**Fig. 6:**
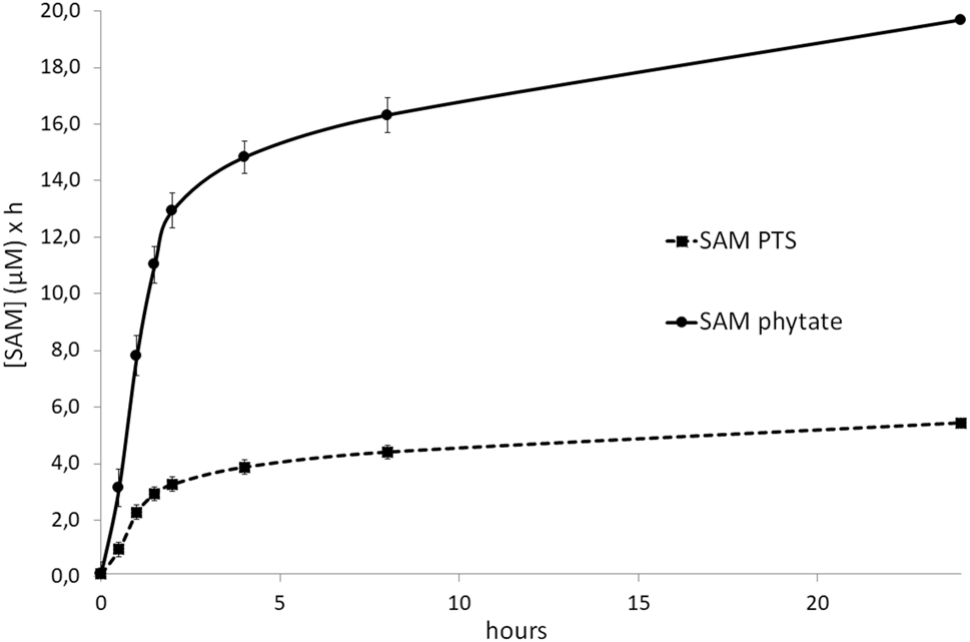
Area under the curve profiles of SAM PTS (dashed line) or SAM phytate (black line) after single oral administration (*N* = 8). Data are mean ± SEM calculated by the propagation error method. Starting from the analysis dataset, the trapezoidal method was adopted for the AUC (at time points 8 h and 24 h) calculation according to the following formula: (*C*_*n*-1_ + *C*_*n*_) × (*T*_*n*_ − *T*_*n*-1_)/2

Results of the analysis of the AUC are shown in Fig. 6. The AUC_0-8_ was 16.31 µM for the SAM phytate group and 4.41 µM for the SAM PTS group. Analogously, the AUC_0-24_ was 19.67 µM for the SAM phytate group and 5.44 µM for the SAM PTS group. The results refer to the AUC calculated on the mean time point. The AUC ratios SAM phytate/SAM PTS at the above-mentioned time point were 3.70 at 8 h (16.31 µM/4.41 µM), and 3.62 at 24 h (19.67 µM / 5.44 µM).

On average, SAM concentrations showed higher *C*_max_ and AUC in the SAM phytate group than in the SAM PTS group, whereas median *T*_max_ was 0.75 in both groups.

### Inferential pharmacokinetic analyses

In addition to the descriptive analyses proposed in the study protocol, to compare the results of the two groups at the *C*_max_ and all the time points after, an inferential analysis was performed. The comparison was done through a non-parametric *t* test (Wilcoxon). The aim of this analysis was to pinpoint possible significant differences at different time points or by sex.

#### All samples

The present analysis refers to the comparison of the baseline-corrected SAM plasma concentrations between the two study groups at all the time points after the study drug administration.

The results are presented in Table 2.

**Table 2:** Wilcoxon test performed on all groups plasma SAM concentration

Time point (hours)	Statistics	SAM phytate (µM) *N* = 8	SAM PTS (µM) *N* = 8	*p* value
0.5	Mean ± SD Median (Q1–Q3)	12.16 ± 2.94 11.06 (10.52–13.0)	3.48 ± 3.07 2.20 (1.13–6.04)	0.0005
1	Mean ± SD Median (Q1–Q3)	6.57 ± 6.48 4.00 (1.03–12.79)	1.74 ± 1.29 1.34 (0.93–2.13)	0.1592
1.5	Mean ± SD Median (Q1–Q3)	6.26 ± 4.34 6.62 (2.41–9.45)	0.99 ± 0.39 0.83 (0.55–1.24)	0.0050
2	Mean ± SD Median (Q1–Q3)	1.39 ± 1.03 0.84 (0.68–2.16)	4.46 ± 0.31 0.42 (0.21–0.67)	0.0068
4	Mean ± SD Median (Q1–Q3)	0.49 ± 0.22 0.52 (0.34–0.66)	0.20 ± 0.32 0.08 (0.00–0.25)	0.0195
8	Mean ± SD Median (Q1–Q3)	0.28 ± 0.20 0.21 (0.17–0.40)	0.14 ± 0.14 0.12 (0.01–0.26)	0.0775
24	Mean ± SD Median (Q1–Q3)	0.20 ± 0.18 0.17 (0.06–0.30)	0.03 ± 0.09 0.00 (0.00–0.00)	0.0049

Data in Table 2 confirm the marked difference observed in Fig. 5. The difference between the two groups was statistically significant at almost all the time points or were borderline significant, except at time point 1 h, where the difference was not statistically significant. That was due to an evident high variability (high SD) in the SAM phytate group at that time point.

#### Analysis by sex

To check for possible gender dependent variables, data were also analyzed separately as male and female group. Tables 3 and 4 report the results of the analysis by sex using the same method adopted for carrying out the analysis in the whole sample. Similar results as in Table 2 were observed in the female and male sub-groups (Tables 3, 4).

**Table 3:** Descriptive and inferential analysis (Wilcoxon test) for the female subgroup

Time point (hours)	Statistics	SAM phytate (µM) *N* = 4	SAM PTS (µM) *N* = 4
0.5	Mean ± SD Median (Q1–Q3)	11.67 ± 2.05 11.38 (10.27–13.0)	2.06 ± 1.42 1.73 (0.96–3.17)
1	Mean ± SD Median (Q1–Q3)	6.28 ± 7.03 4.00 (1.18–11.38)	2.44 ± 1.56 2.13 (1.21–3.67)
1.5	Mean ± SD Median (Q1–Q3)	8.03 ± 2.22 7.56 (0.75–9.45)	1.23 ± 0.21 1.24 (1.09–1.36)
2	Mean ± SD Median (Q1–Q3)	1.31 ± 1.05 0.84 (0.75–1.86)	0.61 ± 0.38 0.67 (0.32–0.90)
4	Mean ± SD Median (Q1–Q3)	0.49 ± 0.30 0.55 (0.27–0.71)	0.23 ± 0.47 0.00 (0.00–0.47)
8	Mean ± SD Median (Q1–Q3)	0.21 ± 0.18 0.20 (0.08–0.33)	0.24 ± 0.12 0.26 (0.16–0.33)
24	Mean ± SD Median (Q1–Q3)	0.10 ± 0.09 0.10 (0.04–0.17)	0.00 ± 0.00 0.00 (0.00–0.00)

**Table 4:** Descriptive and inferential analysis (Wilcoxon test) for the male subgroup

Time point (hours)	Statistics	SAM phytate (µM) *N* = 4	SAM PTS (µM) *N* = 4
0.5	Mean ± SD Median (Q1–Q3)	12.65 ± 3.91 10.83 (10.52–14.7)	4.90 ± 3.82 5.08 (1.60–8.19)
1	Mean ± SD Median (Q1–Q3)	6.85 ± 6.95 6.23 (0.91–12.79)	1.03 ± 0.36 1.07 (0.73–1.34)
1.5	Mean ± SD Median (Q1–Q3)	4.49 ± 5.53 2.41 (0.93–8.05)	0.56 ± 0.14 0.55 (0.44–0.68)
2	Mean ± SD Median (Q1–Q3)	1.47 ± 1.16 1.07 (0.66–2.28)	0.32 ± 0.14 0.33 (0.21–0.42)
4	Mean ± SD Median (Q1–Q3)	0.49 ± 0.17 0.51 (0.35–0.63)	0.16 ± 0.13 0.17 (0.08–0.25)
8	Mean ± SD Median (Q1–Q3)	0.35 ± 0.22 0.28 (0.18–0.51)	0.04 ± 0.08 0.01 (0.00–0.09)
24	Mean ± SD Median (Q1–Q3)	0.30 ± 0.21 0.30 (0.17–0.42)	0.06 ± 0.13 0.00 (0.00–0.13)

**Fig. 7:**
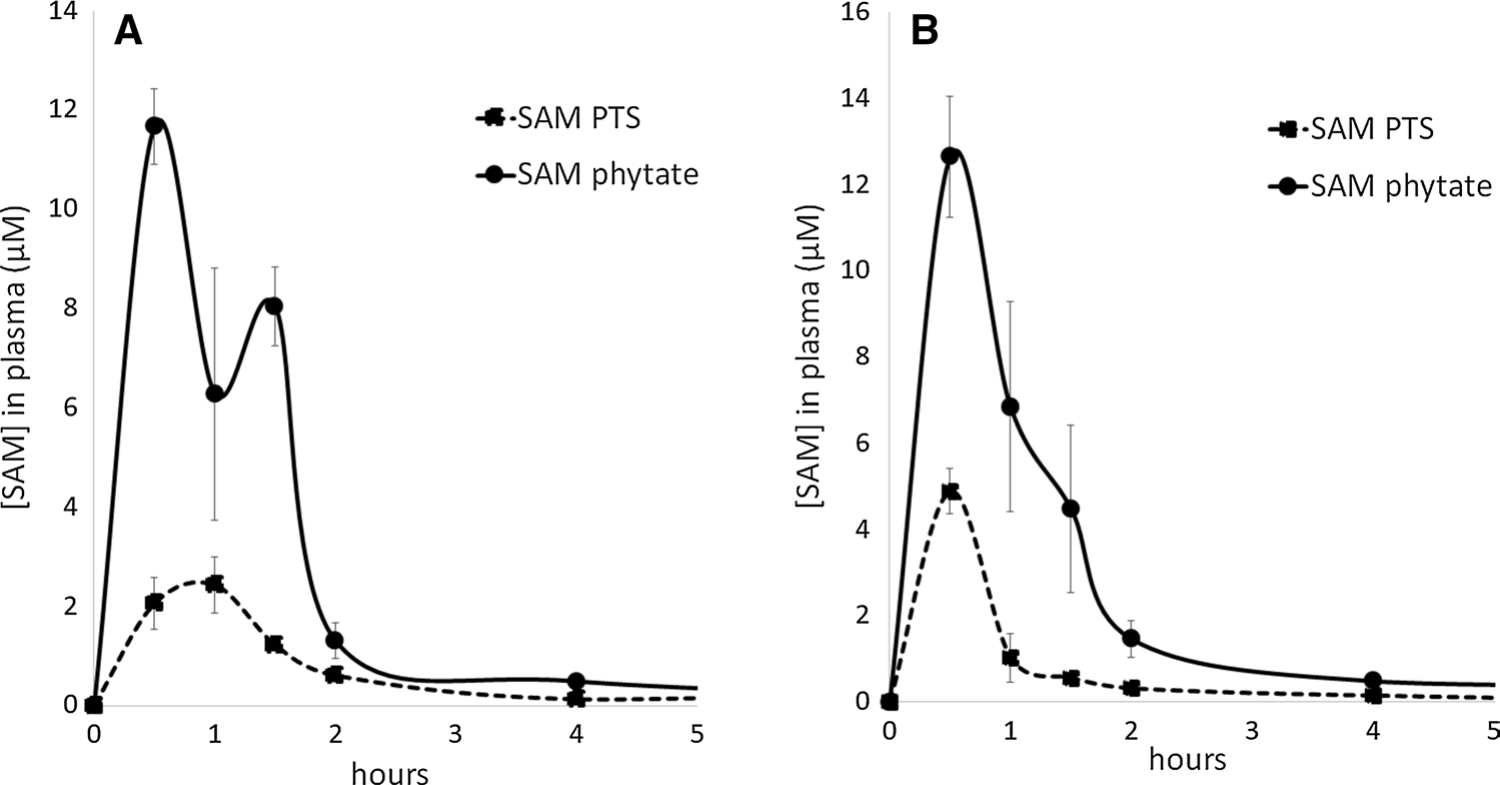
**A** Mean plasma concentration–time profiles of SAM PTS (dotted line) or SAM phytate (black line) after single oral administration in the female group. **B** Mean plasma concentration–time profiles of SAM PTS (dotted line) or SAM phytate (black line) after single oral administration in the male group

The above presented results are more evident in the following Fig. 7A and B, in which the two distributions are plotted.

Rats were administered with equimolar amounts of SAM phytate or SAM PTS via gavage. Plasma was sampled at 0.5, 1, 1.5, 2, 4, 8, and 24 h after dosing, and SAM concentration was determined by HPLC analysis. Data are mean ± SEM of four independent measures for each timepoint.

The plasma concentration between the groups in the whole sample was similar, however, it can be noticed that mean plasma concentration–time profiles between male and female group seems to be slightly different for the SAM PTS group, probably because the expression of enzymes involved in one-carbon metabolism differs between genders (Christensen et al. 2010; Sadre-Marandi et al. 2018). Due to limited number of animals and low statistical power, no statistical analysis was performed on sex differences and data are presented as descriptive data with an explorative aim, as a prompt for future experiments.

## Discussion

The pharmacological potential of SAM urges to find formulations which guarantee stability of the molecule and possibly a better pharmacokinetic profile. In this regard, it has been observed that salts of SAM with strong anions can confer stability to the molecule. In solution, the pH affects the chemical stability of the molecule. Sufficiently acidic media and low temperatures prevent SAM from quickly degrading due to intramolecular attack of the carboxylate anion on the electrophilic γ-carbon which is particularly reactive being nearby the sulfonium ion. When SAM is in the solid crystalline form, its stability is driven by the features of the negative counter ion, i.e., increased steric hindrance exerted by large anionic groups improves stability of the molecule.

The search for possible SAM salts which could be more stable led to the identification of phytate as possible counterion. Phytic acid, or myo-inositol hexaphosphate, is a major form of phosphorylated inositol present in foods such as cereals, nuts, legumes, grains, oilseeds, and some vegetables (Schlemmer et al. 2009). Phytate was long considered to be an antinutrient molecule, as it hampers the intestinal absorption of ions, particularly iron, due to its chelating properties. However, in recent years phytate has been reported to possess various significant health benefits including antioxidant, anticancer, and heart disease prevention activities (Schlemmer et al. 2009). Phytic acid possesses two ionizable hydrogens for each phosphate group, half of which are strongly acidic (pKa 1.1–2.1) and always dissociated in physiological conditions. The other six protons have pKa values ranging from 5 to very weakly acidic protons having a pKa > 10.0 (Costello et al. 1976) allowing phytate to occur as stronger or weaker negatively charged molecule over a wide range of pH values. This feature is very important from a pharmacokinetic point of view because different ionized forms at different pH values can exert different activities (i.e., interaction with proteins, divalent metal ions and, minerals and ionizable biological catalysts) (Humer et al. 2015). Recently, phytate was demonstrated to be a potential absorption enhancer for its ability to selectively act on tight junction proteins of the intestinal mucosa, as shown in a study carried out in a Caco-2 cell model (Fu et al. 2015). It was also reported that oral co-administration of phytate and polyphenolic substances in rats and humans dramatically enhances plasma concentrations of polyphenols and their excretion in the urine (Matsumoto et al. 2007; Xie et al. 2014). Based on these premises, we found of interest to test the stability of a new phytate salt of SAM and to evaluate its pharmacokinetics in vivo.

Preliminary accelerated stability data revealed that SAM is less susceptible to degradation when it is present in the phytate form rather than as the PTS salt. Chromatographic analyses revealed that when the products were stored at 53 °C for 5 days, SAM phytate showed a greater resistance to chemical degradation (Gnosis internal data, N.M. personal communication). SAM phytate confirmed its stability when submitted to an ICH stability protocol at 25 °C and 60% RH lasting 6 months (Fig. 3).

The product was hence administered to rats to evaluate potential differences in the pharmacokinetic characteristics of the molecule. Rats were divided into two groups, one of which was administered the SAM phytate salt, the other one a standard formulation containing an equimolar amount of SAM PTS, a commercial product which is widely available on the market. Both products were found to be very well tolerated and no adverse effects were registered. After a single oral administration, mean plasma concentration profiles of SAM PTS or SAM phytate differed significantly, with phytate salt being absorbed better than the PTS salt as clearly evident in Fig. 5. The outcome of the statistical comparisons showed a significant difference between the two SAM salts at all-time points, as shown in Table 2, except for the 1 h time point, whose significance is above the threshold, due undoubtedly to a high intrinsic variability in the data. SAM plasma concentrations showed significantly higher rate (*C*_max_) and extent (AUC_0-t_) after single dose of SAM phytate than the SAM PTS formulation, whereas *T*_max_ of the two products was not significantly different, indicating that the speed of absorption is not affected, but that SAM phytate is more bioavailable compared to the PTS salt. These results support the previously described in vitro evidence which indicate that phytate may enhance intestinal permeability by transiently loosening the tight junctions, thus favoring molecules passage through the intestinal epithelium (Fu et al 2015).

It is of interest to note that a very recent publication by Cameron and coworkers (Cameron et al. 2020) observed a similar effect for a new patented formulation of SAM, the MSI-195 formulation, in a pharmacokinetic study in humans. In this product, SAM is associated to propyl gallate, which was found to enhance systemic absorption of SAM probably by facilitating paracellular transport, which is the same mechanism proposed for phytate (Fu et al 2015).

An analysis was also performed to highlight possible differences linked to sex, by comparing the descriptive analyses between male and female groups. The results reported in Tables 3 and 4 show that the SAM phytate group had a higher plasma concentration in comparison to what observed for the SAM PTS group at all the considered time points both for male and for female animals, but due to the limited number of animals it was not possible to evaluate the statistical significance between male and female animals. Interestingly, the same evaluation was carried out by Cameron and coworkers (Cameron et al. 2020), which observed a lack of consistent directionality to the gender effects, hence they concluded that more studies involving a wider number of subjects are needed to get more reliable data. On the other hand, lack of gender effect was already observed for healthy volunteers supplemented with oral SAM (Yang et al. 2009).

In conclusion, our results demonstrate that the new formulation of SAM phytate has a good chemical stability and a better pharmacokinetic profile in rats compared to the standard PTS salt widely used in nutraceutical and pharmaceutical preparations both for animal and human health. These results open to the perspective of the use of new salts of SAM endowed with a better stability or pharmacokinetic properties.
